# Successive Trajectory Privacy Protection with Semantics Prediction Differential Privacy

**DOI:** 10.3390/e24091172

**Published:** 2022-08-23

**Authors:** Jing Zhang, Yanzi Li, Qian Ding, Liwei Lin, Xiucai Ye

**Affiliations:** 1 School of Computer Science and Mathematics, Fujian University of Technology, Fuzhou 350118, China; 2 Fujian Provincial Key Laboratory of Big Data Mining and Applications, Fuzhou 350118, China; 3 Department of Computer Science, University of Tsukuba, Tsukuba 305-8577, Japan

**Keywords:** trajectory publishing, differential privacy, prediction, sensitivity, Markov chain

## Abstract

The publication of trajectory data provides critical information for various location-based services, and it is critical to publish trajectory data safely while ensuring its availability. Differential privacy is a promising privacy protection technology for publishing trajectory data securely. Most of the existing trajectory privacy protection schemes do not take into account the user’s preference for location and the influence of semantic location. Besides, differential privacy for trajectory protection still has the problem of balance between the privacy budget and service quality. In this paper, a semantics- and prediction-based differential privacy protection scheme for trajectory data is proposed. Firstly, trajectory data are transformed into a prefix tree structure to ensure that they satisfy differential privacy. Secondly, considering the influence of semantic location on trajectory, semantic sensitivity combined with location check-in frequency is used to calculate the sensitivity of each position in the trajectory. The privacy level of the position is classified by setting thresholds. Moreover, the corresponding privacy budget is allocated according to the location privacy level. Finally, a Markov chain is used to predict the attack probability of each position in the trajectory. On this basis, the allocation of the privacy budget is further adjusted and its utilization rate is improved. Thus, the problem of the balance between the privacy budget and service quality is solved. Experimental results show that the proposed scheme is able to ensure data availability while protecting data privacy.

## 1. Introduction

Location-based service (LBS) has become increasingly popular in people’s daily lives due to the proliferation of mobile devices [1]. At present, LBS has covered all aspects of national economy and social life, such as navigation, query and recommendation of interest points, takeout, check-in, social networking [2], etc. Moreover, the implementation of LBS depends on published trajectory data [3]. However, when releasing trajectory data, there is a probability of being attacked by attackers, resulting in the disclosure of users’ trajectory information. The disclosure of trajectory information may lead to the exposure of more personal privacy information, so trajectory privacy has become one of the most important privacies of people.

Traditional trajectory privacy protection technologies include K-anonymity, encryption and differential privacy [4]. The K-anonymity model and its derivative model provide a means of quantitative evaluation, which makes different types of schemes comparable, but cannot provide strict mathematical proof [5]. Meanwhile, the security depends on the background knowledge grasped by the attacker. In addition, cryptography-based privacy protection methods can provide strict protection on data confidentiality, but their disadvantages and challenges lie in weak scalability and low implementation efficiency [6]. This is mainly because the current homomorphic encryption mechanisms inevitably have large computational complexity overhead. The emergence of differential privacy technology makes up for the above problems effectively. It hides sensitive raw data by attaching a noise value that obeys a certain distribution to the raw data. On the one hand, the differential privacy model makes the maximum assumption about the attacker’s ability, and does not depend on the background knowledge the attacker has mastered [7]. On the other hand, the differential privacy model is built on a solid mathematical basis, and gives a quantitative model of the degree of privacy leakage, which is simple to implement and efficient to calculate. However, existing studies on trajectory differential privacy protection still have problems in the following three aspects:

(1) The existing trajectory privacy protection mechanism does not take into account the problem of excessive overhead of real-time sensitivity calculation. It is difficult to obtain accurate sensitivity of each position in the trajectory, although the amount of calculation is reduced offline.
(2) The impact of semantic location on trajectory is not considered in the previous scheme. Semantic location is likely to increase the risk of user privacy information disclosure. For example, users’ preferences and economic level can be inferred according to the frequency of users’ access to certain semantic location points.
(3) In the publishing process of the differential privacy trajectory data set, the allocation of the privacy budget is one of the key factors determining the final amount of noise added. If the privacy budget is not allocated properly, it can cause serious waste and add too much overall noise. However, the current method of privacy budget allocation still stays at average allocation or simple balance allocation, and there is still a certain degree of waste. How to design a more reasonable way of privacy budget allocation according to the characteristics of trajectory data sets is still lacking in relevant research.

If the sensitivity can only be calculated in real time, the calculation cost is too large, which will increase the time cost of the scheme and reduce the operation efficiency. In this paper, a sensitivity map is defined so that the sensitivity of each position point of trajectory can be queried offline. If the impact of semantic location is not taken into account, it is likely to increase the risk of privacy leakage. For example, a user’s trajectory is between home and school every day. School is a special semantic location. After acquiring the user’s trajectory, the attacker can infer his occupation or even economic status easily. This paper takes into account the impact of semantic location on user location sensitivity to improve the privacy protection effect. In addition, if the allocation of privacy budget is not reasonable, the added noise will be too large or too small. This can result in reduced data availability or insufficient privacy. Therefore, the allocation method of privacy budget is improved in this paper. A semantics- and prediction-based differential privacy protection scheme for trajectory data (SPDP) is proposed in this paper. The contributions are summarized as follows:

(1) A sensitivity map is defined so that the sensitivity of the current position can be accurately confirmed even offline. Thus, the computational overhead is reduced and the operating efficiency of this scheme is improved. The differentiation protection mechanism of location privacy based on a sensitivity map is designed. By allowing users to customize the sensitivity of semantic locations, the privacy budget can be tailored to further improve its utilization.
(2) The differentiation protection mechanism of location privacy based on semantic location is designed. Considering the influence of semantic location sensitivity, sensitivity is determined by the number of trajectories containing the node prefix and semantic sensitivity. The privacy levels are divided according to the location sensitivity. Then the sensitivity ratio and privacy levels are used to allocate the privacy budget of each location to further improve its utilization.
(3) A privacy budget adjustment algorithm based on a Markov chain is proposed. After the privacy budget is allocated based on sensitivity and privacy level, the attack probability of the nodes in the prefix tree is calculated by using the property of the Markov process. Then, the sensitivity and privacy level are adjusted by attack probability, so as to adjust the allocation of privacy budget and make the allocation of privacy budget more reasonable.

The rest of the article is organized as follows: the related work is given in Section 2; the preliminaries are given in Section 3; the privacy protection method is designed in Section 4; the simulation analysis is discussed in Section 5; and finally, the conclusion is given in Section 6.

## 2. Related Work

The relevant technologies involved in this paper include trajectory differential privacy protection [8,9,10] and location recommendation mechanism [11,12,13]. Therefore, the typical methods of trajectory differential privacy protection and location recommendation mechanism are analyzed, respectively.

Due to the gradual increase of location service applications, the research on privacy protection of location trajectory data has become a hot research topic. In recent years, the differential privacy model based on false data technology has been rapidly applied to protect the privacy of data release after being proposed. This model realizes privacy protection by adding noise to real data sets [14]. In data release, differential privacy realizes different privacy protection degrees and data release accuracy by adjusting privacy parameter ε. Generally speaking, the higher the value of ε is, the lower the degree of privacy protection is, and the higher the accuracy of published data sets is. Differential privacy is mainly realized through a noise mechanism. The first universal differential privacy mechanism is the Laplace mechanism proposed in [15], which is mainly aimed at numerical query. For non-numerical queries, the exponent mechanism is proposed in [16], which is the second universal mechanism to realize differential privacy.

In the privacy protection of trajectory data set release, the prefix method based on the differential privacy model is proposed for the first time in [17]. This method uses a hierarchical framework to construct a prefix tree, divides the trajectories with the same prefix into the same branch of the tree, and realizes differential privacy by adding noise to the node count. However, as the tree grows, the prefix will form a large number of leaf nodes, resulting in too much noise and reducing the accuracy of the published data set. Later, on the basis of prefix method, location trajectory and check-in frequency are used to set thresholds in [18], so as to classify the level of location sensitivity. Then, the corresponding privacy budget is allocated according to the sensitivity, which makes the allocation of privacy budget more reasonable and reduces the amount of noise data.

The work [19] proposes the method of merging similar trajectories. By dividing the trajectory coverage area into grids, the trajectory position points falling into the same grid are represented by the center points of the grid, thus improving the counting value of position points greatly. In [20], the regional division is improved by adopting a multi-level grid model to divide position points at different speeds in the trajectory according to different granularity, so as to maintain the original sequence information of the trajectory to the maximum extent. However, these methods have the problem of low data availability due to excessive information loss rate, and fail to fully consider the semantic location information of users, resulting in semantic inference attacks [21], which leads to the disclosure of users’ sensitive privacy.

Published trajectory data can be used in various location services. Location recommendation service in LBS is frequently used. For example, Nur [22] presents a new problem of user identification of top-K social space co-participation location selection (SSLS) in social graphs. Two exact solutions and two approximate solutions are developed to solve this NP-hard problem. Thus, the best set of K positions can be selected for the user from a large number of candidate positions. Location recommendation methods can be divided into three categories generally: content-based recommendation system, collaborative filtering recommendation and mixed recommendation [23]. A content-based recommendation system mainly selects items with high similarity to them as recommendations according to the items users like. Collaborative filtering technology determines a group of recommender users with similar behaviors according to the evaluation behavior of the target users, and takes the evaluation of the recommender users on the project as the recommendation value of the target users. Mixed recommendation is mainly to solve the deficiency of single recommendation technology. Different recommendation technologies can be combined according to different mixing strategies to complete the recommendation.

Lian first proposes a collaborative filtering algorithm based on implicit feedback and content perception [24], which gives a lower preference value to the locations that users have not visited, and a higher preference value to the locations that users have visited according to their historical access frequency. Then, Lian combines the matrix factor decomposition method and puts forward the improved schemes Geo MF [25] and Geo MF++ [26], which improve the accuracy of the recommendation system effectively. In recent years, with the development of deep learning theory, neural network technology has also been used to solve the problem of location recommendation [27,28,29]. Shyamali [30] proposes the fault tolerance technology of the relevant sensitive random logic circuit to reduce the system error. Lalli [31] reduces operational risk by training four neural networks to detect and handle errors before they cause harm. However, the technology needs a lot of data support. In addition, the above recommendation schemes only focus on the recommendation effect and ignore the user’s privacy and security issues. The lack of protection of trajectory data may cause the disclosure of user’s privacy information easily.

The existing studies on trajectory differential privacy protection do not take into account the impact of semantic features on trajectory, and the privacy budget allocation is not precise enough. In addition, the existing location recommendation mechanisms ignore the privacy protection of user data. Therefore, a semantics- and prediction-based differential privacy protection scheme for trajectory data is proposed in this paper. The semantic sensitivity and the Markov technology are introduced to improve the utilization rate of the privacy budget. Meanwhile, the location recommendation mechanism is combined with the differential privacy technology to protect the security of the trajectory data while ensuring the location recommendation effect.

## 3. Preliminaries

**Figure 1:**
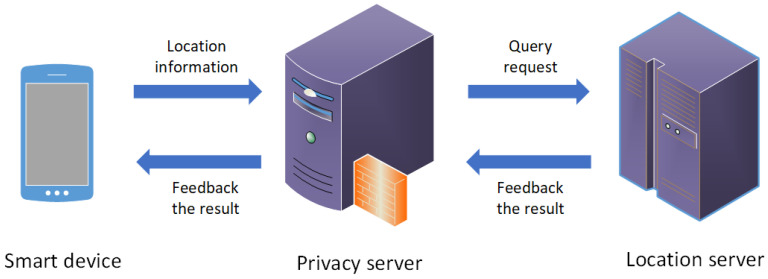
The system model of SPDP.

The system model of semantics and prediction based differential privacy protection scheme for trajectory data (SPDP) is presented in Figure 1. The system model consists of three parts: mobile smart device, privacy server and location server. Among them, privacy server is a trusted third party anonymous server. This paper focuses on the privacy protection of the system, so it ignores the details of the internal network connection. The location information to be protected is the trajectory data published by the mobile smart device, including the user check-in time, location identification (ID), longitude and latitude. These assumptions are used in most previous works, such as [7,17,18]. In addition, the SPDP scheme proposed in this paper uses differential privacy technology, prefix tree structure, Markov chain and so on to protect the trajectory data. Therefore, the definitions of related concepts are quoted and designed. The detailed definitions involved are shown below.

**Definition** **1.** 
ε ***-Differential Privacy*** *[14]. Given a query algorithm* $M:D\to R^{d}$ *that supports a random mechanism, if for any data set* D *and its adjacent data set* $D^{\prime }$ *, algorithm* M *satisfies Formula (1) for any output* O *, then the random algorithm* M *satisfies* ε *-differential privacy.*

(1) $$\mathrm{Pr}\left(M\left(D\right)\in O\right)\leq e^{\varepsilon }\cdot \mathrm{Pr}\left(M\left(D^{\prime }\right)\in O\right)$$

*There is only one record difference between adjacent data sets, that is,*$\|D-D^{\prime }\|{}_{1}=1$. ε*is the privacy budget, which determines the degree of privacy protection and the accuracy of released data sets. The lower the privacy budget is, the closer the probability ratio of algorithm*M*outputting the same result on*D*and*$D^{\prime }$*is to 1, and the higher the degree of privacy protection is, the lower the accuracy of the corresponding published data set is. When*ε*= 0,*M*will output the result with the same probability distribution on*D*and*$D^{\prime }$*, and the degree of privacy protection will reach the highest at this moment, but the published data will not reflect any useful information.*

**Definition** **2.**  ***Global Sensitivity*** *[16]. For any query function* $f:D\to R^{d}$ *, the global sensitivity of* f *is*

(2) $$\Delta f=max_{D,D^{\prime }}\|f\left(D\right)-f{\left(D^{\prime }\right)\|}_{1}$$

*Global sensitivity is the maximum range of output value variation of a particular query function* f *on all possible adjacent datasets* D *and* $D^{\prime }$ *, and its measure is the* L1 *distance between the two.*

**Definition** **3.**  ***Laplacian Mechanism****[8]. For any function*f*on data set*D*, if the output result of function*f*satisfies Equation (3), then the random algorithm*M*satisfies*ε*-differential privacy.*

(3) $$M\left(D\right)=f\left(D\right)+\mathrm{Lap}{\left(\frac{\Delta f}{\varepsilon }\right)}^{d}$$

*where,*Δf*is the sensitivity of the query function. The location parameter of the Laplace distribution is 0, and the scale parameter is*$\frac{\Delta f}{\varepsilon }$.

**Definition** **4.**  ***Trajectory Prefix****[17]. A trajectory*$S=s_{1}\to s_{2}\to \cdots \to s_{\left|S\right|}$*is a prefix of a trajectory*$T=t_{1}\to t_{2}\to \cdots \to t_{\left|T\right|}$*, denoted by*S≼T*, if and only if*|S|≤|T|*and*∀1≤i≤|S|*,*$s_{i}=t_{i}$.

**Figure 2:**
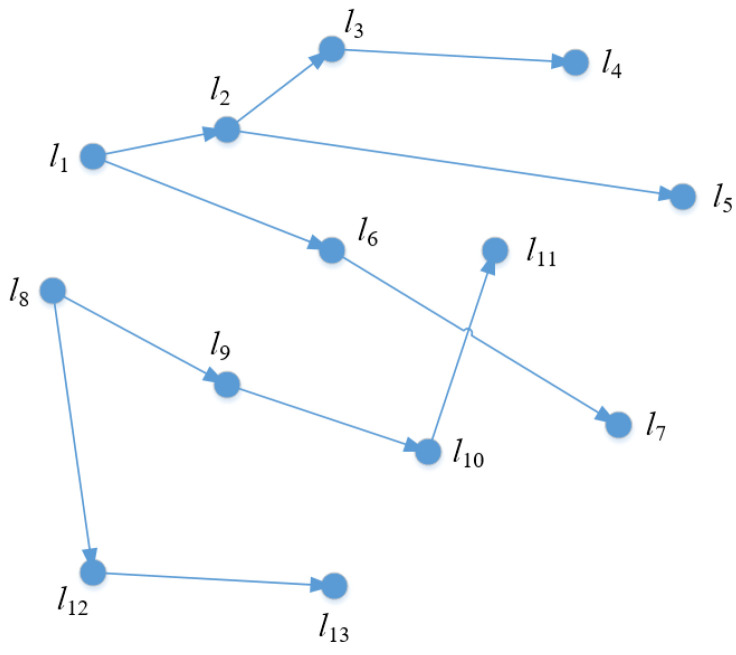
One trajectory of user u.

For example, a trajectory and the corresponding trajectory sequence of user u are shown in Figure 2 and Table 1. For trajectory 1: $\;l_{1}\to l_{2}\to l_{3}\to l_{4}$, it can be seen that $l_{1},l_{1}\to l_{2}$, $l_{1}\to l_{2}\to l_{3}$ and $l_{1}\to l_{2}\to l_{3}\to l_{4}$ are their prefixes, but $l_{2}\to l_{3}$ is not a prefix.

**Definition** **5.**  ***Prefix Tree****[17]. A prefix tree*TT*of a trajectory database*D*is a triplet*TT=(V,E,Root(TT))*, where*V*is the set of nodes labeled with locations, each corresponding to a unique trajectory prefix in*D*;*E*is the set of edges, representing transitions between nodes;*Root(TT)∈V*is the virtual root of*TT*. The unique trajectory prefix represented by a node*v∈V*, denoted by prefix* (v,TT)*, is an ordered list of locations starting from*Root(TT)*to*v.

**Figure 3:**
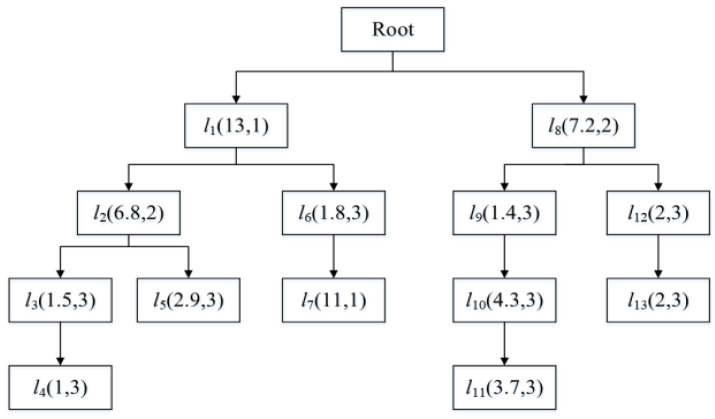
The prefix tree structure of user u.

Each node v∈V of TT keeps a doublet in the form of $\left(S_{i},pl_{i}\right)$, where $S_{i}$ is the location sensitivity, and $pl_{i}$ is the privacy level of the location. Figure 3 illustrates the prefix tree of the sample database in Table 1, where each node v is labeled with its location, sensitivity and privacy level.

**Definition** **6.**  ***Semantic location**. Semantic location refers to the location that conforms to the characteristics of semantic location type, denoted as*SL*. In this paper, semantic location types are divided into 10 categories according to geographic tags, including science, education and culture, catering, leisure and entertainment, medical care and so on. Semantic locations can be obtained from map information. Each semantic location has a certain semantic sensitivity, and will affect the location sensitivity within a certain range. Therefore, each location*$l_{i}$*has a certain semantic sensitivity, denoted as*$Sem_{i}$.

**Definition** **7.**  ***Sensitivity**. The check-in times of user* u *at location* $l_{i}$ *can indicate the user’s preference for this location. It is assumed that the more times users check in, the higher the preference degree of users for this location. Attackers can easily master users’ preferences by calculating check-in statistics of specific locations, so users’ privacy is vulnerable to leakage. To solve this problem, this paper defines the sensitivity of the user’s check-in location:* $S_{i}=\alpha_{i}+Sem_{i}$ *. Where,* $\alpha_{i}$ *represents the check-in times of user* u *at position* $l_{i}$ *, and* $Sem_{i}$ *represents the semantic sensitivity of user at position* $l_{i}$ *. As shown in Figure 3 and Table 2. The user’s check-in times* $\alpha_{i}$ *and semantic sensitivity* $Sem_{i}$ *are combined as the location sensitivity* $S_{i}$ *of node* $l_{i}$ *. The more times a user checks in to a location, the more sensitive that location is.*

**Definition** **8.**  ***Privacy Level**. The location privacy level is defined as* pl=r (r=1,2,⋯,n) *in this paper. It is determined by the sensitivity of user* u *to the location. Three privacy levels are set in this paper, namely insensitive, normal and sensitive. Then the thresholds are set for position sensitivity. When sensitivity reaches the thresholds, the privacy level of this location changes.*

It is defined as the highest privacy level when pl=1 in this paper. As sensitivity increases, the privacy level of a location decreases. In other words, the position is most sensitive when pl=1, and the position is less sensitive when the value of pl is larger. If pl is small, the sensitivity of the position is relatively high, that is, the more sensitive position $l_{i}$ is, the less weight it will have. Therefore, the privacy budget allocated to location $l_{i}$ is small. In differential privacy protection, the smaller the privacy budget allocated to position $l_{i}$, the greater the added noise and the higher the privacy protection intensity.

For example, divide the privacy level for the trajectory example of user u shown in Figure 2 and Table 1. Suppose the position is least sensitive when the assumed sensitivity is less than 5. Then assume that the threshold interval is 5, and when the sensitivity exceeds the threshold, the privacy level will change. When the sensitivity exceeds 10, the privacy level is the highest and the location is the most sensitive. Accordingly, the privacy level division of the trajectory example is obtained, as shown in Table 3.

**Definition** **9.**  ***Markov Process*** *[32]. Assume that the time parameter set of random process* $X=\left\{X_{t},t\in T\right\}$ *is* T={0,1,⋯} *and the state space* E *is discrete,* $E=\left\{i_{0},i_{1},i_{2},\cdots \right\}$ *. For any*  t∈R *,* $i_{0},i_{1},i_{2},\cdots \in E$ *, then:*

(4) $$P\left(X_{t}=i_{t}|X_{t-1}=i_{t-1},X_{t-2}=i_{t-2},\cdots ,X_{0}=i_{0}\right)=P\left(X_{t}=i_{t}|X_{t-1}=i_{t-1}\right)$$

*If the random process* X *satisfies Equation (4), the random process is a Markov process. Where,* $\left\{X_{t}=i\right\}$ *represents the state of random process* X *at time*  t *is* i *. The property of Markov processes is that the future state is only related to the present state, not to the past state.*

## 4. Semantics- and Prediction-Based Differential Privacy Protection Scheme for Trajectory Data (SPDP)

**Figure 4:**
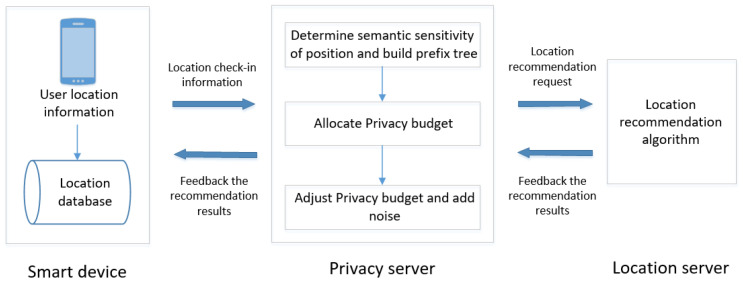
The framework of SPDP.

The specific process of the semantics- and prediction-based differential privacy protection scheme for trajectory data (SPDP) proposed in this paper is shown in Figure 4.

Step 1. Sensitivity processing based on semantic location: Allocate different privacy budgets for different semantic locations, determine the semantic sensitivity of the location through the generated semantic sensitivity map, and obtain the location sensitivity and privacy level by combining the check-in times of the location.

Step 2. Privacy budget allocation based on prefix tree: A single location satisfying ε-differential privacy cannot ensure trajectory privacy security. Therefore, the user trajectory is transformed into a prefix tree structure to ensure that the trajectory meets ε-differential privacy, and the privacy budget is allocated according to the sensitivity of the location.

Step 3. Privacy budget adjustment based on Markov chain: The attack probability of the location is predicted by a Markov chain, and the allocated privacy budget is adjusted according to the attack probability to further improve its utilization rate.

Step 4. Location recommendation under differential privacy protection: Add corresponding noise to the location, and reflect the validity and availability of trajectory data under differential privacy protection through the recommendation effect of location recommendation service.

### 4.1. Sensitivity Processing Based on Semantic Location

Not only semantic locations directly connected to sensitive locations are sensitive. From the perspective of random disturbance distribution, those semantic locations close to sensitive locations still have the risk of exposing sensitive locations even if they are not connected to sensitive locations directly. Therefore, certain semantic sensitivity should also be assigned. This paper considers the global connectivity between location points and radiates the semantic sensitivity of semantically sensitive locations to nearby nodes according to the distance and access degree.

**Figure 5:**
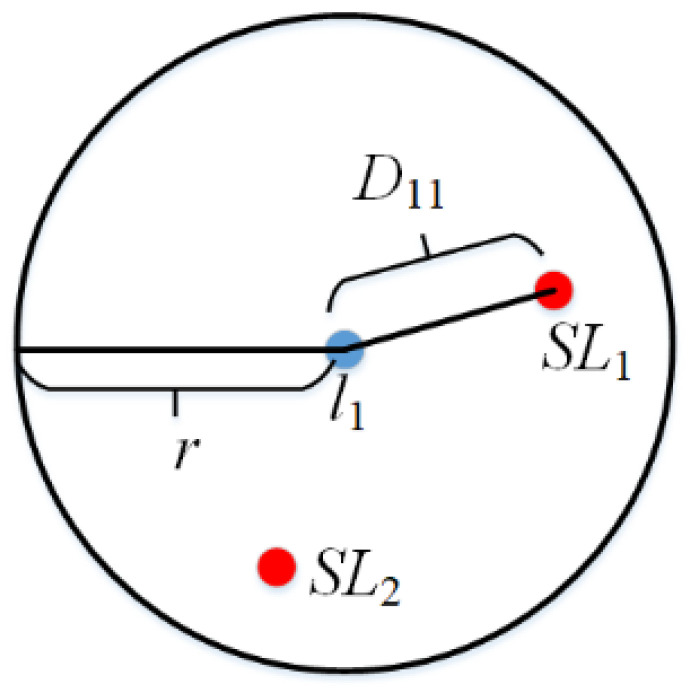
Semantic sensitivity of position $l_{i}$.

As shown in Figure 5, the semantic location node set A with a privacy level near any location $l_{i}$ is first obtained. Then, the map is transformed into an undirected graph. According to the distance and access degree, the equivalent distance between any location $l_{i}$ and semantic location $SL_{j}$ is $D_{ij}=d_{SL_{j}}\left(c_{j}-1\right)$. Where, $d_{SL_{j}}$ is the Euclidean distance between $l_{i}$ and $SL_{j}$, and $c_{j}$ is the number of nodes traversed by the shortest path between the two nodes. Finally, the semantic sensitivity of semantic location radiation in A of any location $l_{i}$ is obtained, as shown in Equation (5).

(5) $$Sem_{i}=\sum_{SL_{j}\in A}\frac{r-D_{ij}}{r}\cdot Sem_{SL_{j}}$$

where, $Sem_{i}$ represents the semantic sensitivity of location $l_{i}$. $A=\left\{SL|d_{SL_{j}}<r\right\}$, r indicates the threshold set by the user.

For the convenience of calculation, we use this paper grid map. Then, the semantic sensitivity of each region in the map is calculated using the above process, and the semantic sensitivity map $map_{sen}$ is generated.

In Algorithm 1, the check-in times $\alpha_{i}$ and semantic sensitivity $Sem_{i}$ of each node point in data set T are calculated firstly, and the two are combined as the sensitivity $S_{i}$ of node (1–6 lines of Algorithm 1). Lines 7–12 of Algorithm 1 divide privacy levels according to node sensitivity. Based on the experimental data, this paper divides the privacy level into three categories. When pl=1, the position is the most sensitive, when pl=2, it is classified as normal, and when pl=3, it is classified as insensitive. If the sensitivity of the node is less than 10, the privacy level is set to level 3. If the sensitivity is between 10 and 50, the privacy level is set to level 2. If sensitivity is greater than or equal to 50, the privacy level is set to level 1. Finally, a prefix tree is constructed and the sensitivity map ${map}_{sen}$ is generated according to the sensitivity and privacy level of nodes (13–15 lines of Algorithm 1).
**Algorithm 1: Sensitivity Processing Algorithm Based on Semantic Location****Input**: User check-in location data set T **Output**: Sensitivity map $map_{sen}\left(l_{i},S_{i},pl_{i}\right)$, prefix tree TT begin   1: $l_{i}\leftarrow T,\;S_{i}\leftarrow \emptyset ,\;pl_{i}\leftarrow \emptyset$;   2: **for** every position $l_{i}$ in T **do**
  3:  $\alpha_{i}\leftarrow T$;   4:   $A\leftarrow \left\{SL_{j}|d_{SL_{j}}<r\right\}$;   5:   $Sem_{i}\leftarrow \underset{SL_{j}\in A}{\sum }\frac{r-D_{ij}}{r}\cdot Sem_{SL_{j}}$;   6:   $S_{i}\leftarrow \alpha_{i}+Sem_{i}$;   7:  **if**  $S_{i}<10\;$
  8:    $pl_{i}=3$;   9:  **else** $10\leq S_{i}<50\;$
  10:    $pl_{i}=2$;   11:   **else** $S_{i}\geq 50\;$
  12:    $\;pl_{i}=$1;   13:   $\mathrm{TT}\leftarrow l_{i}\left(S_{i},pl_{i}\right)$;   14:  **end for**
  15: **return** ${map}_{sen}\left(l_{i},S_{i},pl_{i}\right)$, TT end

### 4.2. Privacy Budget Allocation Based on Prefix Tree

Because the root node in the prefix tree is not the actual check-in location, the root node does not consume the privacy budget. The privacy budget allocation scheme in this paper is mainly divided into two steps: the privacy budget allocation of each trajectory subsequence and the privacy budget allocation of each child node on the trajectory subsequence. Firstly, the average sensitivity of each trajectory subsequence is calculated to calculate the access probability of each subsequence. Then, the privacy budget is assigned to the trajectory subsequence according to the access probability. Since the higher the access probability, the higher the sensitivity, the allocated privacy budget should be inversely proportional to the access probability. Secondly, the privacy budget is allocated to each node according to the proportion of each node’s privacy level in the sum of the privacy level of each trajectory subsequence. Finally, because part of the location points appear in multiple trajectory subsequences, the repeated privacy budget is merged. The privacy budget allocation algorithm based on location sensitivity is shown as follows:

In Algorithm 2, the privacy budget (lines 1–4 of Algorithm 2) is first assigned to the trajectory subsequence. The average sensitivity of each trajectory in dataset T is calculated. Then, the access probability of each trajectory is calculated according to the proportion of sensitivity, and the privacy budget is allocated according to the inverse relationship between the access probability and the privacy budget. In lines 5–7 of Algorithm 2, the privacy budget is allocated to each location in the trajectory according to the location’s privacy level, and finally, the privacy budget of the location in multiple trajectories is combined.
**Algorithm 2: Privacy Budget Allocation Algorithm Based on Sensitivity****Input**: Privacy budget *ε*, prefix tree TT **Output**: Trajectory set TB after allocating privacy budget Begin   1: **for** every trajectory $T_{i}$ in TT **do**;   2:  $S_{T_{i}}\leftarrow \frac{\sum_{j=1}^{M}S_{lj}}{M_{i}}$;   3:  $P_{T_{i}}\leftarrow \frac{S_{T_{i}}}{S_{T_{1}}+\cdots +S_{T_{N}}}$;   4:  $\varepsilon_{T_{i}}\leftarrow \varepsilon \cdot \frac{\frac{1}{P_{T_{i}}}}{\sum_{i=1}^{N}\frac{1}{P_{T_{i}}}}$;   5:  **for** every position $l_{j}$ in $T_{i}$
**do**;   6:   $\varepsilon_{l_{ij}}\leftarrow \varepsilon_{T_{i}}\cdot \frac{p_{l_{j}}}{\sum_{j=1}^{M}p_{l_{j}}}$;   7:  $\varepsilon_{j}\leftarrow \overset{N}{\underset{i=1}{\sum }}\varepsilon_{l_{ij}}$;   8:  **end for**
  9: **end for**
  10: **return** TB
end

### 4.3. Privacy Budget Adjustment Based on Markov Chain

A trajectory consists of a series of position points that are continuous. The property of the Markov chain corresponds to the trajectory, that the next position depends only on the previous position. The two most important components of the Markov chain are the initial state probability distribution and state transition matrix.

Assume that the possible location set generated by the user at the moment is $L^{\left(t-1\right)}=l_{1}^{\left(t-1\right)},l_{2}^{\left(t-1\right)},\cdots ,l_{m}^{\left(t-1\right)}$, and its probability value is $P^{\left(t-1\right)}=p_{1}^{\left(t-1\right)},p_{2}^{\left(t-1\right)},\cdots ,p_{m}^{\left(t-1\right)}$. That is the initial state probability distribution. Suppose there are n possible positions for a user’s trajectory, namely $l_{1},l_{2},\ldots ,l_{n}$. The state transition probability from position $l_{i}$ to position $l_{j}$ is denoted as $P\left(l_{i}\to l_{j}\right)$, then matrix P is formed, which is called state transition probability matrix.

(6) $$P=\left[\begin{array}{ccc} P_{11} & \cdots & P_{1n}\\ \vdots & \ddots & \vdots \\ P_{n1} & \cdots & P_{nn} \end{array}\right]$$

Then, the state transition probability matrix is used to calculate the possible position at time t as $L^{\left(t\right)}=l_{1}^{\left(t\right)},l_{2}^{\left(t\right)},\cdots ,l_{m}^{\left(t\right)}$, and its probability value is $P^{\left(t\right)}=p_{1}^{\left(t\right)},p_{2}^{\left(t\right)},\cdots ,p_{m}^{\left(t\right)}$, where $P^{\left(t\right)}=P^{\left(t-1\right)}P$, is the attack probability of the possible position at time t.

Assume that an attacker’s attack starts at the initial position of the trajectory and continues in the direction of the trajectory. The property of Markov process is used to calculate the attack probability of nodes in the prefix tree, and the sensitivity is adjusted by calculating the probability, so as to adjust the allocated privacy budget. The privacy budget adjustment algorithm based on Markov is shown in Algorithm 3.

In Algorithm 3, the access probability of each trajectory is firstly calculated, and then the access probability of each position in the trajectory is calculated as the initial probability state distribution (lines 1–8 of Algorithm 3). Then, the state transition matrix is calculated according to the proportion of check-in times in the data set, so as to obtain the attack probability at time t (lines 9–11 of Algorithm 3). Finally, sensitivity and privacy level are adjusted linearly according to the attack probability, so as to adjust the privacy budget (lines 12–15 of Algorithm 3).
**Algorithm 3: Privacy Budget Adjustment Algorithm Based on Markov****Input**: Trajectory set TB after allocating privacy budget **Output**: Trajectory set TC after adjusting privacy budget Begin   1: **for** every trajectory $T_{i}$ in TB **do**;   2:  $S_{T_{i}}\leftarrow \frac{\sum_{j=1}^{M}S_{lj}}{M_{i}}$;   3:  $P_{T_{i}}\leftarrow \frac{S_{T_{i}}}{S_{T_{1}}+\cdots +S_{T_{N}}}$;   4:  **for** every position $l_{j}$ in $T_{i}$
**do**;   5:   $p_{lj}\leftarrow P_{T_{i}}\cdot \frac{S_{lj}}{\sum_{j=1}^{M}S_{lj}}$;   6:   $P^{\left(t-1\right)}\leftarrow p_{lj}$;   7:  **end for**
  8: **end for**
  9: $\;p_{ij}\leftarrow \mathrm{TB}$;   10: $P=\left[\begin{array}{ccc} P_{11} & \cdots & P_{1n}\\ \vdots & \ddots & \vdots \\ P_{n1} & \cdots & P_{nn} \end{array}\right]$;   11: $P^{t}=P^{\left(t-1\right)}\cdot P$;   12: **for** every position $l_{j}$ in TB **do**
  13:  $S_{l_{j}}^{\prime }\leftarrow S_{l_{j}}+10\times P^{t}$;   14:  $pl_{l_{j}}^{\prime }\leftarrow S_{l_{j}}^{\prime }$;   15:  $\varepsilon_{l_{j}}^{\prime }\leftarrow pl_{l_{j}}^{\prime }$;   16: **end for**
  17: **return** TC end

### 4.4. Location Recommendation under Differential Privacy Protection

Through the previous three sections, the privacy budget assigned by the user for each location is available. Then, the Laplace mechanism is used to add the corresponding noise to the sensitivity of the position to change the privacy level of the position in this paper. As the location privacy level changes, it is difficult for an attacker to discover a user’s true preference for the location.

After the location privacy level is changed, the interest score of user u on location l is calculated by Equation (7). Where, $S_{u,l}^{\prime }$ and $w_{score}^{\prime }$ represent the position sensitivity and position score weight after adding noise, respectively:

(7) $$IG_{u,l}=S_{u,l}^{\prime }\times w_{score}^{\prime }$$

Position l is most sensitive when pl is minimal. However, when the location score is calculated, the weight of the location will increase as the privacy level of the location increases. Therefore, Equation (8) is used in this paper to calculate the score weight of the position.

(8) $$w_{score}=\frac{pl_{n-r+1}}{\sum_{r}^{n}pl_{r}}$$

Since location sensitivity is used as location score directly, the score difference between locations will be too large, affecting the accuracy of the results. Therefore, $IG_{u,l}$ is normalized to obtain the normalized location score $IGN_{u,l}$, and then the scoring matrix $Matrix_{IGN}$ of users and locations is constructed, $IGN_{u,l}$ is shown as follows:

(9) $$IGN_{u,l}=\frac{IG_{u,l}-min\left(IG_{u,l}\right)}{max\left(IG_{u,l}\right)-IG_{u,l}}$$

After obtaining score matrix $Matrix_{IGN}$, the Pearson correlation coefficient is used to calculate users’ similarity sim(u,v), and user similarity matrix $Matrix_{sim}$ is constructed, where sim(u,v) represented the similarity between user u and user v.

(10) $$sim\left(u,v\right)=\frac{\sum_{l\in l\left(u,v\right)}\left(IGN_{u,l}-\bar{IGN_{u,l}}\right)\left(IGN_{v,l}-\bar{IGN_{u,l}}\right)}{\sqrt{\sum_{l\in u}{\left(IGN_{u,l}-\bar{IGN_{u,l}}\right)}^{2}}\sqrt{\sum_{l\in v}{\left(IGN_{v,l}-\bar{IGN_{u,l}}\right)}^{2}}}$$

where, l(u,v) represents the common check-in location set of user u and user v, and $\bar{IGN_{u,l}}$ represents the average location score of user u. Finally, according to the user similarity matrix $Matrix_{sim}$, n users with the highest similarity to the target user are regarded as similar users. In addition, the locations of similar users are set and the locations not visited by target users are arranged in descending order of score, and the first n locations are recommended to target users. The location recommendation algorithm is as follows.

Assume that an attacker’s attack starts at the initial position of the trajectory and continues in the direction of the trajectory. The property of the Markov process is used to calculate the attack probability of nodes in the prefix tree, and the sensitivity is adjusted by calculating the probability, so as to adjust the allocated privacy budget. The privacy budget adjustment algorithm based on Markov is shown in Algorithm 4.
**Algorithm 4: Location Recommendation Algorithm****Input**: Trajectory set TC after adjusting privacy budget **Output**: Location recommendation set LR Begin   1: **for** every position $l_{i}$ in TC **do**;   2:  $S_{u,l}^{\prime }\leftarrow S_{u,l}+\mathrm{Lap}{\left(\frac{\Delta f}{\varepsilon_{i}}\right)}^{d}$;   3:  $pl_{r}^{\prime }\leftarrow S_{u,l}^{\prime }$;   4:  ${w_{score}}^{\prime }\leftarrow \frac{pl_{n-r+1}}{\sum_{r}^{n}pl_{r}}$;   5:  $IG_{u,l}\leftarrow S_{u,l}^{\prime }\times w_{score}^{\prime }$;   6:  $IGN_{u,l}\leftarrow \frac{IG_{u,l}-min\left(IG_{u,l}\right)}{max\left(IG_{u,l}\right)-IG_{u,l}}$;\\ Normalize for $IG_{u,l}$
  7:  **for** every user $v_{j}$ in TC **do**
  8:   $sim\left(u,v_{j}\right)\leftarrow \frac{\sum_{l\in l\left(u,v_{j}\right)}\left(IGN_{u,l}-\bar{IGN_{u,l}}\right)\left(IGN_{v_{j},l}-\bar{IGN_{u,l}}\right)}{\sqrt{\sum_{l\in u}{\left(IGN_{u,l}-\bar{IGN_{u,l}}\right)}^{2}}\sqrt{\sum_{l\in v_{j}}{\left(IGN_{v_{j},l}-\bar{IGN_{u,l}}\right)}^{2}}}$;   9:    Arrange $sim\left(u,v_{j}\right)$ in descending order, take   the top-*n* users in $v_{j}$;   10:   **for** the $l_{k}$ in top-*n* users that are not accessed by   the target user **do**
  11:    Arrange $IGN_{v_{j},l_{k}}$ in descending order, take   the top-*n* locations in $l_{k}$
  12:   **end for**
  13:  **end for**
  14: **end for**
  15: **return** top-*n* $l_{k}$
end

In Algorithm 4, noise is first added to the sensitivity and privacy level of the location (lines 1–3 of Algorithm 4). Then score weight and interest score are calculated and normalized (lines 5–6 of Algorithm 4). Lines 7–9 of Algorithm 4 calculate the similarity between users and take the first *n* users with the highest similarity. Finally, the position with the highest interest score among the first *n* locations that are not visited by similar users is selected for recommendation (lines 10–15 of Algorithm 4).

## 5. Performance Evaluations

### 5.1. Experimental Environment

In this section, the scheme in this paper is simulated and analyzed. The simulation platform is realized by using PYTHON language. The computer platform used for the experiment is an Intel Core I5-6300HQ computer with 8 GB memory and Windows 10 64-bit computer. In this experiment, the real public location data set Gowalla [33] is used. In order to obtain better experimental results, check-in records of 2000 active users in one year are selected. The Gowalla’s data format is shown in Table 4, which contains the user’s unique identification, check-in time, and location information.

### 5.2. Feasibility Analysis

**Figure 6:**
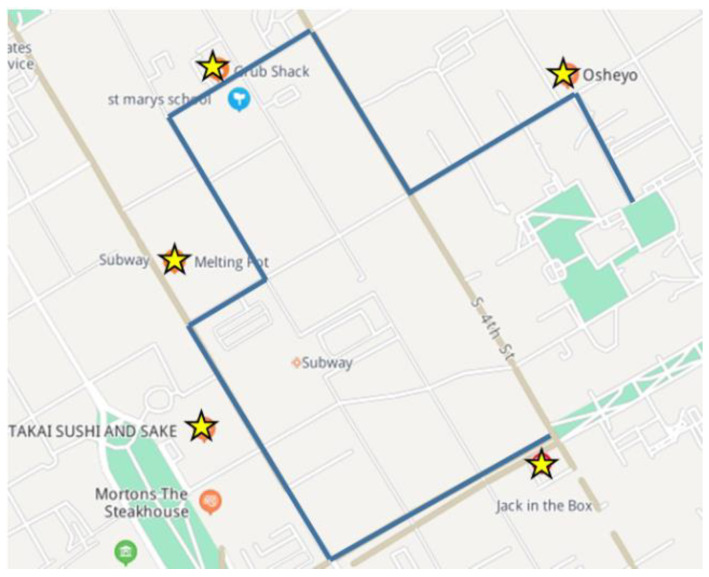
One example of the recommendation result.

Firstly, the feasibility of the scheme is analyzed. As shown in Figure 6, five recommendation positions with n=5 are generated to visually display the effect of the recommendation algorithm. Where, the blue line segment represents the user’s trajectory, and the yellow marks represent the recommended locations of the user. It can be seen from Figure 6 that the recommended positions are similar to the positions through which the user trajectory passes, and there is no overlap with the trajectory. Therefore, the SPDP proposed in this paper is feasible.

### 5.3. Evaluation Indicators

In order to evaluate the application effect of the scheme proposed in this paper in the location recommendation service, the evaluation indexes commonly used in the recommendation system were selected in the experiment: *Precision*, *Recall* and F-Score [34]. Then, in order to analyze the efficiency, the variation of the algorithm operation time is shown when the number of location recommendations is different. Finally, in order to evaluate the privacy protection degree after adding noise, the ratio of location sensitivity is statistically compared, which means the ratio of the number of locations with different privacy levels to the total number of locations.

*Precision* and *Recall* are defined as Equation (11) and Equation (12), respectively, where U represents the user set, LR represents the length of recommendation list, R(u) represents the recommended location set of user u, and T(u) represents the interest location set of user u in the test set.

(11) $$Precision=\frac{1}{\left|U\right|}\sum_{u\in U}\frac{\left|R\left(u\right)\cap T\left(u\right)\right|}{LR}$$

(12) $$Recall=\frac{1}{\left|U\right|}\sum_{u\in U}\frac{\left|R\left(u\right)\cap T\left(u\right)\right|}{T\left(u\right)}$$

F-Score represents overall recommendation quality by weighting *Precision* and *Recall*. The comprehensive recommendation effect of the proposed scheme can be evaluated by comparing F-Score. The higher the F-Score is, the higher the recommendation quality is. The definition is shown in Equation (13):

(13) $$F-\mathrm{Score}\;=\frac{2\times Precision\times Recall}{Precision+Recall}$$

The location sensitivity ratio is defined in Equation (14), where $\left|pl_{r}\right|$ represents the total number of user’s locations with privacy level of $pl_{r}$, $\left|l_{u}\right|$ represents the total number of user’s locations, and |U| represents the total number of users.

(14) $$R=\frac{\sum_{u\in U}\frac{\left|pl_{r}\right|}{\left|l_{u}\right|}}{\left|U\right|}$$

### 5.4. Experimental Results

In order to prove the effectiveness of SPDP in this paper, it is compared with BOSD in reference [18] and UD of uniform distribution in reference [17]. In this experiment, a total of three levels of location privacy are set. The initial sensitivity for this paper is set to 10. When the sensitivity is less than 10, the location privacy level is 3, that is, the location is not sensitive. Then, the threshold interval is initially set to 40. When the sensitivity is between 10 and 50, the location privacy level is 2, meaning that the sensitivity of the location is normal. When the sensitivity exceeds 50, the maximum privacy level of the location is 1, that is, the location is a sensitive location. Top-*n* (*n* = 5) is used to obtain the set of candidate locations with the highest similarity.

**Figure 7:**
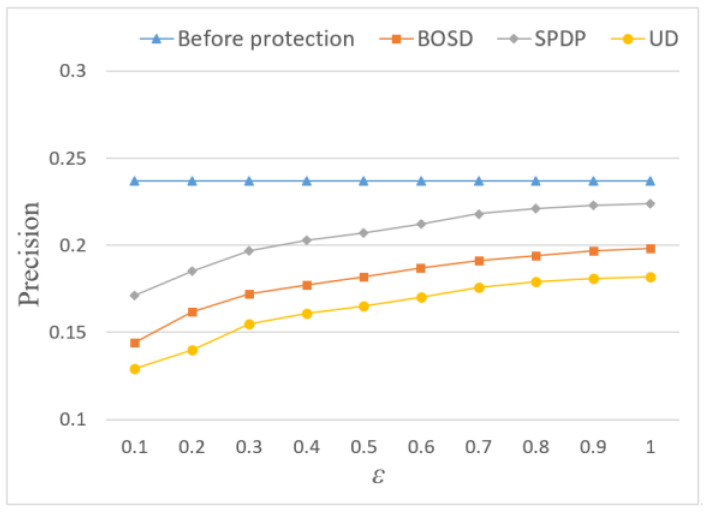
Influence of privacy budget ε on Precision.

**Figure 8:**
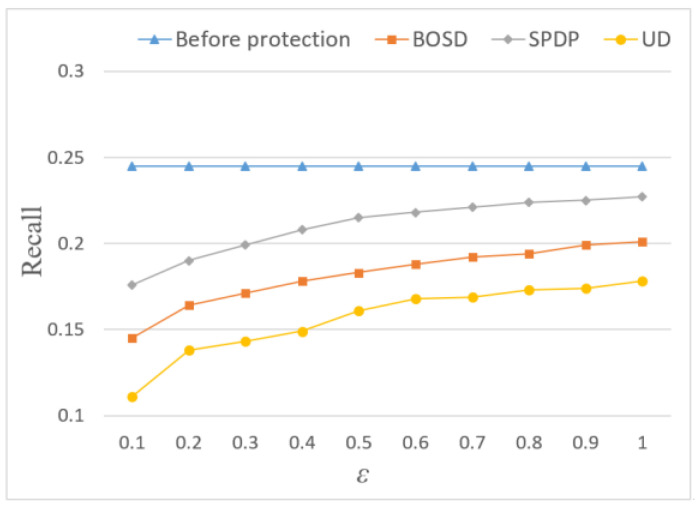
Influence of privacy budget ε on Recall.

In terms of *Precision* and *Recall*, the recommendation quality of the three methods is inferior to that before adding noise. It can be seen from Figure 7 and Figure 8 that adding Laplace noise will reduce the effect of location recommendation. This is because the noise changes the statistical characteristics of the original trajectory data set and produces certain errors, thus affecting the result of location recommendation. However, the *Precision* and *Recall* of the SPDP are still better than BOSD scheme and UD scheme, reaching 22.4% and 22.7%, respectively. This is because this paper considers the influence of semantic location and further adjusts the allocation of location privacy budget through Markov chain, which makes the recommendation effect of this scheme better.

**Figure 9:**
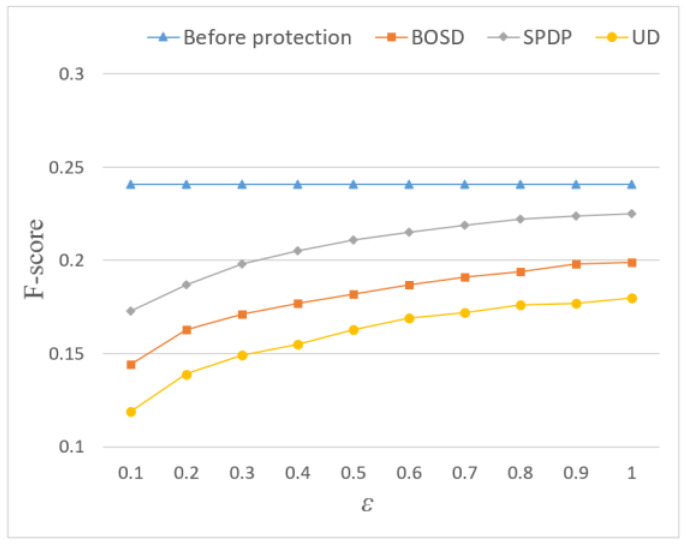
Influence of privacy budget ε on F-Score.

The influence of ε on F-Score is shown in Figure 9. The SPDP proposed by this paper is superior to BOSD and UD in terms of comprehensive recommendation quality, with F-Score reaching 22.5%, but there is still some recommendation quality loss. In this paper, the privacy budget allocation method based on location sensitivity keeps the frequency characteristics of users’ original trajectory access, and considers the influence of semantic location and users’ preference for location. It can better maintain the similarity between positions and reduce the similarity error caused by noise addition. However, due to the sparse check-in data of users, the experimental results will be affected to some extent.

**Figure 10:**
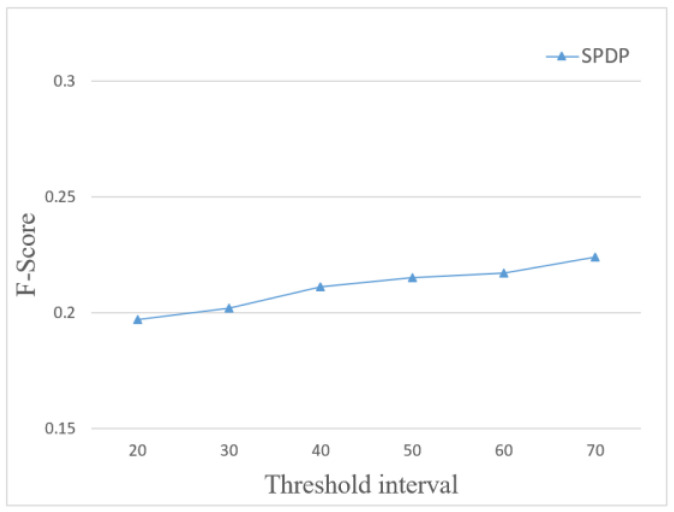
Influence of threshold interval on F-Score.

Since the location privacy level is determined by setting a threshold based on the sensitivity of the location, the final result is also affected by the setting of the threshold range. Therefore, the relationship between the comprehensive recommendation quality and the threshold range is compared when the total location privacy levels are 3 and the privacy budget is 0.5. The threshold interval increases from 20, as shown in Figure 10.

It can be seen from Figure 10 that, as the threshold increases, the threshold range between each privacy level increases, and the overall recommendation quality also improves. This is because, as the threshold range increases, the number of high privacy locations gradually decreases, so the noise added to them also decreases. However, a decrease in the number of locations with higher privacy levels means a decrease in the intensity of privacy protection. Therefore, it is necessary to set a reasonable threshold range to achieve a certain balance between the quality of location recommendation and privacy protection.

**Figure 11:**
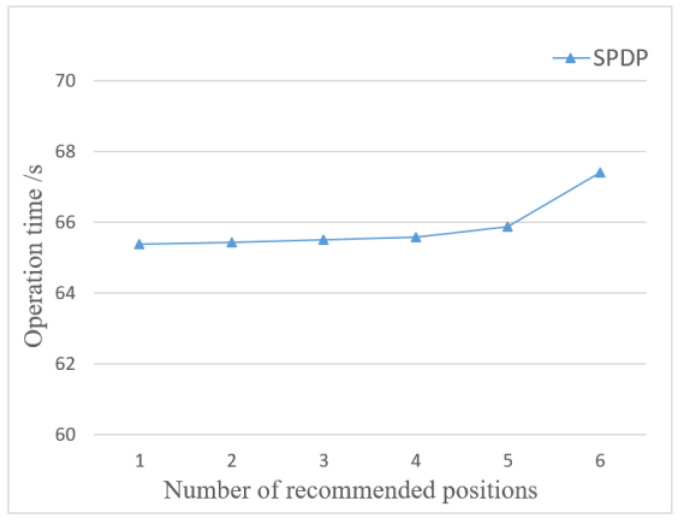
Influence of number of recommended positions on operation time.

Figure 11 shows the influence of the number of recommended locations on the operation time. The privacy budget is set to 0.5. As can be seen from Figure 11, the operation time is proportional to the number of recommended locations, that is, the more the number of recommended locations, the more operation time. This is because the more the number of recommended locations, the more time it takes to calculate the sensitivity of location points, and the more time it takes to calculate the privacy level of each location point. So, the higher the number of recommended locations, the longer the operation time.

**Figure 12:**
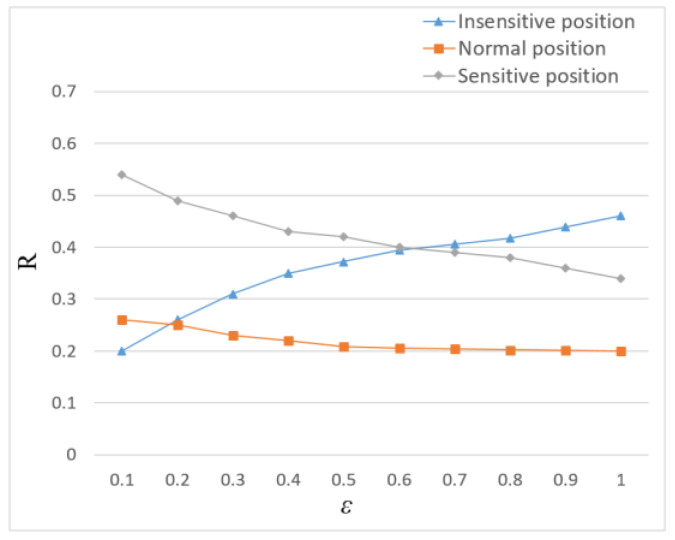
Location sensitivity ratio *R* under different privacy budget ε.

Figure 12 illustrates the impact of privacy budgets on location sensitivity. As shown in Figure 12, with the increase of privacy budget, the added noise gradually decreases, and the proportion of the sensitive position and normal position gradually decreases, while the proportion of the insensitive position gradually increases. This is because the scheme based on sensitivity partition in this paper allocates the budget according to the privacy level and adds more noise to the position with high sensitivity. As the number of sensitive locations increases, it is difficult for the attacker to distinguish the real sensitive locations, thus reducing the probability of identification. Therefore, better privacy protection for sensitive positions on the trajectory can be provided by this scheme.

### 5.5. Theoretical Analysis

**Definition** **10.**  ***Sequential Composition*** *[7]. Given a query algorithm* $\;M:D\to R^{d}$ *that supports a random mechanism, if for any data set* D *and its adjacent data set* $D^{\prime }$ *, algorithm* M *satisfies Formula (1) for any output* O *, then the random algorithm* M *satisfies* ε *-differential privacy.*

Sequential composition means that given database D and n random algorithms $\left\{A_{1},A_{2},\cdots ,A_{n}\right\}$, if each algorithm $A_{i}$ acting on data set D satisfies $\varepsilon_{i}-DP$, then the sequential sequence group on D satisfies $\left(\overset{n}{\underset{i=1}{\sum }}\varepsilon_{i}\right)-DP$. Sequential composition indicates that when multiple algorithm sequences act on a data set at the same time, the final privacy budget is the sum of each algorithm’s privacy budget.

**Definition** **11.**  ***Parallel Composition*** *[7]. Divide a database* D *into* n *disjoint sets* $\left\{D_{1},D_{2},\cdots ,D_{n}\right\}$ *, and apply a random algorithm* $\left\{A_{1},A_{2},\cdots ,A_{n}\right\}$ *, respectively, on each set, and* $A_{i}$ *satisfies* $\varepsilon_{i}-DP$ *. Then, the parallel sequence combination on*  D *satisfies* $\left(max\varepsilon_{i}\right)-DP$ *. The parallel composition indicates that if multiple algorithms operate on disjoint subsets of a data set, the final privacy budget is the maximum of each algorithm’s privacy budget.*

**Theorem** **1.**  *Given the total privacy budget ε, Algorithm 3 ensures ε-differential privacy.*

**Proof** **of** **Theorem** **1.**  In the process of building the noise prefix tree, the noise prefix tree TC is constructed with an easy to understand query model. Consider the height of a prefix tree. It is known that all nodes on the same layer of the prefix tree contain a disjoint set of trajectories. According to Definition 11, the total privacy budget required by each layer is limited by the worst case, that is, $\bar{\varepsilon }=\frac{\varepsilon }{h}$ [17]. Allocating privacy budgets at different levels follows Definition 10. Since there are at most h levels, the total privacy budget required to construct a noisy prefix tree is $\leq h\times \bar{\varepsilon }=\varepsilon$. □

## 6. Conclusions

In order to protect the trajectory data security of data release, a semantics- and prediction-based differential privacy protection scheme for trajectory data is proposed in this paper. In this scheme, trajectory sequences are stored by a prefix tree structure, and the privacy level of the location is divided by check-in statistics combined with the influence of semantic location. Then, the privacy budget is allocated according to the privacy level, and further adjusted through the Markov chain. The appropriate differential privacy noise is added to the user’s check-in position sensitivity, and the position sensitivity level is changed to achieve the effect of privacy protection. By analyzing the experimental results of real location data sets, the proposed scheme can protect the trajectory privacy of users and reduce the impact of differential privacy noise on the quality of service effectively.

The scheme proposed in this paper is based on the centralized differential privacy, which requires that the third-party service providers are completely trusted and will not actively steal or passively leak users’ private information. However, in practical applications, it is impossible to find an absolutely secure third-party service provider. Therefore, the local differential privacy model will be introduced to better resist attacks on third-party servers in future research work, so as to achieve better privacy protection effects.

## Figures and Tables

**Table 1:** The trajectory sequence of user u.

The Serial Number	The Trajectory	The Serial Number	The Trajectory
1	$l_{1}\to l_{2}\to l_{3}\to l_{4}$	4	$l_{8}\to l_{9}\to l_{10}\to l_{11}$
2	$l_{1}\to l_{2}\to l_{5}$	5	$l_{8}\to l_{12}\to l_{13}$
3	$l_{1}\to l_{6}\to l_{7}$	6	$l_{12}\to l_{13}$

**Table 2:** The position sensitivity of user u.

Location	$S_{i}$	$\alpha_{i}$	$Sem_{i}$
$l_{1}$	13	3	10
$l_{2}$	6.8	2	4.8
$l_{3}$	1.5	1	0.5
$l_{4}$	1	1	0
$l_{5}$	2.9	1	1.9
$l_{6}$	1.8	1	0.8
$l_{7}$	11	1	10
$l_{8}$	6.2	2	4.2
$l_{9}$	1.4	1	0.4
$l_{10}$	4.3	1	3.3
$l_{11}$	3.7	1	2.7
$l_{12}$	2	2	0
$l_{13}$	2	2	0

**Table 3:** The privacy level and sensitivity of user u.

Location	$S_{i}$	$\alpha_{i}$	$Sem_{i}$	$pl_{i}$
$l_{1}$	13	3	10	1
$l_{2}$	6.8	2	4.8	2
$l_{3}$	1.5	1	0.5	3
$l_{4}$	1	1	0	3
$l_{5}$	2.9	1	1.9	3
$l_{6}$	1.8	1	0.8	3
$l_{7}$	11	1	10	1
$l_{8}$	6.2	2	4.2	2
$l_{9}$	1.4	1	0.4	3
$l_{10}$	4.3	1	3.3	3
$l_{11}$	3.7	1	2.7	3
$l_{12}$	2	2	0	3
$l_{13}$	2	2	0	3

**Table 4:** Partial data from Gowalla dataset.

User ID	Check-In Time	Latitude of Check-In Location	Longitude of Check-In Location	Location ID
0	2010-10-19T23:55:27Z	30.2359091167	−97.7951395833	22847
0	2010-10-18T22:17:43Z	30.2691029532	−97.7493953705	420315
0	2010-10-17T23:42:03Z	30.2557309927	−97.7633857727	316637
0	2010-10-17T19:26:05Z	30.2634181234	−97.7575966669	16516
0	2010-10-16T18:50:42Z	30.2742918584	−97.7405226231	5535878
0	2010-10-12T23:58:03Z	30.261599404	−97.7585805953	15372

## Data Availability

The data that support the findings of this study are available from the corresponding author upon reasonable request.
